# Organ preservation for T2-T3 rectal cancer: opportunistic or planned strategy

**DOI:** 10.18632/oncotarget.26916

**Published:** 2019-05-28

**Authors:** Jéan-Pierre Gérard, Nicolas Barbet, Karen Benezery

**Affiliations:** Department of Radiation Oncology, Centre Antoine Lacassagne, Nice Côte-d’Azur University, Nice 06189, France

**Keywords:** rectal cancer, conservative treatment, contact X-ray brachytherapy, clinical complete response, non operative modality

The standard treatment of T2-3 Nx M0 rectal adenocarcinoma is proctectomy with total mesorectal excision (TME) often combined with neoadjuvant chemoradiotherapy (nCRT). Such radical surgery can be responsible for significant morbidity or suboptimal bowel function when anterior resection is performed. To improve these outcomes organ preservation using a non-operative modality (NOM) is gaining interest in clinical research.

Historically organ preservation was initiated with two different approaches. In the 1960s Papillon used contact X-ray brachytherapy 50 kV (CXB) to control T1 tumor [1]. The lyon R96-02 phase III trial demonstrated that adding CXB to external beam radiotherapy (EBRT) for T2 T3 rectal cancer significantly increased the rate of clinical complete response (cCR), sphincter and organ preservation [2]. In the 1970s Habr Gama initiated a conservative approach using nCRT with a “watch and Wait” strategy in case of cCR [3]. This strategy is presently the world reference. Habr Gama using an extensive CRT regiment achieves a cCR rate of 72% and 63% respectively in T2 and T3 and a local recurrence rate of 8% and 40% [3]. Most of the other experiences are collected in the IWWD (international W-W data base) [4] reporting more than 1000 patients. When treating locally advanced rectal cancer (LARC) with nCRT the rate of cCR is between 15% to 40% and local recurrence between 20 to 35%.

To achieve rectal preservation a cCR must be assessed after nCRT. This requires an intense surveillance protocol combining digital rectal examination, endoscopy and MRI. There is no consensus on the definition of cCR and a grey zone exists between cCR and partial response (PR) called near clinical complete response (ncCR). As the definition of ncCR is still more uncertain than cCR, the extension of the surveillance period from 6 to 12 weeks demonstrated that it could change ncCR in cCR and facilitate a NOM decision [5]. An observation period between 3 to 6 months appears recommended to optimally select patients candidate for NOM. The IWWD showed that the rate of organ preservation at the end of this long period is between 15% to 40% with a risk of local recurrence (sometimes called “regrowth”) close to 20% after NOM.

These suboptimal results can be explained by two main arguments:

- Adenocarcinoma of the rectum is a radioresistant tumor. The model of Appelt is confirming this concept [6]. When correlating radiation dose escalation with pCR rate a dose of 92 Gy appears necessary to sterilize only 50% of T3 tumor. Such a high dose cannot be reached with EBRT alone without severe radiation toxicity. A possible technique to increase safely the RT dose is CXB endocavitary approach. In 2009 of a new CXB machine (Papillon 50^TM^) made possible a renaissance of this technique. Five centers in France and UK using this new system showed that a boost dose of 90 Gy in 3 fractions could be safely delivered with nCRT [7–9].
- Tumor selection. It is a well-known radiobiology concept that the tumor volume is a key parameter for irradiation to achieve sterilization. Rectal adenocarcinomas exceeding 5 cm in diameter are unlikely to be controlled with CRT. The ESMO guidelines [10] recommend to separate the early tumors: T2–T3a-b from the LARC T3c-d or T4, the latest being poor candidate for organ preservation. With such a selection the five CXB centers have been able to treat more than 300 patients. The chance of cCR after a combination of nCRT with CXB boost of 90 Gy in 3 fractions was above 70% and the risk of local failure did not exceed 15%. With tumors T2 -T3a < 3.5 cm diameter this treatment can be initiated using CXB first followed by CRT. In such a situation a cCR is seen in 50% of cases on week 4 after two CXB fractions and a cCR (or ncCR) in more than 90% 2 months after treatment start. With patient selection and safe CXB dose escalation, it is possible to propose this treatment option for rectal cancer as it is standard for squamous cell carcinoma of the anus. This approach can be called “ planned” organ preservation for early rectal cancer [9].

In summary two different strategies can be discussed. The first is using nCRT for LARC. Tumor response is assessed during a long time period of 3 to 6 months and the chance of cCR (or ncCR) at the end does not exceed 40%. This surveillance time is a period of uncertainty for the doctors and anxiety for the patients. Such approach could be called an opportunistic strategy aiming at uncertain probability of organ preservation. The second one is using selection of early rectal cancer and CXB intensified radiotherapy. In this case the probability of cCR (or ncCR), especially when CXB is the initial treatment, is close to 90% and can be rapidly assessed at week 4 or month 2 after treatment initiation. This strategy can be proposed as a planned protocol with high chance of NOM and acceptable low risk of local recurrence. In all cases a close surveillance is mandatory to salvage potential local recurrence.

The ongoing OPERA phase III trial (ID-RCB =2014-A01851-46) is aiming at comparing these two strategies. First preliminary data on 140 patients should be available in 2020. Other ongoing phase III trials using different intensification treatments called total neoadjuvant treatment (TNT) will help to better define the role of NOM in rectal cancer. The ultimate goal is to improve the quality of life of patients by avoiding TME surgery. If proven successful, such a conservative approach will be a real breakthrough in the treatment of rectal cancer.
